# Challenges and Opportunities for the Future of Brain-Computer Interface in Neurorehabilitation

**DOI:** 10.3389/fnins.2021.699428

**Published:** 2021-07-02

**Authors:** Colin Simon, David A. E. Bolton, Niamh C. Kennedy, Surjo R. Soekadar, Kathy L. Ruddy

**Affiliations:** ^1^Trinity College Institute of Neuroscience and School of Psychology, Trinity College Dublin, Dublin, Ireland; ^2^Department of Kinesiology and Health Science, Utah State University, Logan, UT, United States; ^3^School of Psychology, Ulster University, Coleraine, United Kingdom; ^4^Clinical Neurotechnology Laboratory, Neurowissenschaftliches Forschungszentrum, Department of Psychiatry and Neurosciences, Charité – Universitätsmedizin Berlin, Berlin, Germany

**Keywords:** brain-computer interface, stroke, neurorehabilitation, transcranial magnetic stimulation, neurofeedback

## Abstract

Brain-computer interfaces (BCIs) provide a unique technological solution to circumvent the damaged motor system. For neurorehabilitation, the BCI can be used to translate neural signals associated with movement intentions into tangible feedback for the patient, when they are unable to generate functional movement themselves. Clinical interest in BCI is growing rapidly, as it would facilitate rehabilitation to commence earlier following brain damage and provides options for patients who are unable to partake in traditional physical therapy. However, substantial challenges with existing BCI implementations have prevented its widespread adoption. Recent advances in knowledge and technology provide opportunities to facilitate a change, provided that researchers and clinicians using BCI agree on standardisation of guidelines for protocols and shared efforts to uncover mechanisms. We propose that addressing the speed and effectiveness of learning BCI control are priorities for the field, which may be improved by multimodal or multi-stage approaches harnessing more sensitive neuroimaging technologies in the early learning stages, before transitioning to more practical, mobile implementations. Clarification of the neural mechanisms that give rise to improvement in motor function is an essential next step towards justifying clinical use of BCI. In particular, quantifying the unknown contribution of non-motor mechanisms to motor recovery calls for more stringent control conditions in experimental work. Here we provide a contemporary viewpoint on the factors impeding the scalability of BCI. Further, we provide a future outlook for optimal design of the technology to best exploit its unique potential, and best practices for research and reporting of findings.

Brain-computer interfaces (BCIs) are hailed as a promising approach to overcome paralysis by translating brain signals from movement intentions into computerised or motorised feedback. They can be used to restore, replace, enhance, supplement, or improve the natural output of the central nervous system (CNS), hence, providing opportunities for motor rehabilitation from a range of conditions including spinal cord injury, traumatic brain injury and stroke. Following a stroke, approximately 77% of survivors are left with some degree of upper limb impairment (Nakayama et al., 1994; Lawrence et al., 2001), which is a key factor in preventing their engagement in normal activities of daily living and rendering them dependant on caregivers. Rehabilitation approaches that actively promote intensive and prolonged functional use of the paretic limb result in the largest gains in movement capability (Veerbeek et al., 2014). However, the gold standard approaches such as constraint-induced movement therapy (CIMT) require the patient to be capable of producing a sufficient level of functional movement in order to partake (Kwakkel et al., 2015). This prevents participation for those who are more severely impaired, or patients in the early weeks after brain injury who have not yet regained any function. With mounting recent evidence indicating that early intervention is crucial to harness the brain’s endogenous recovery processes (Stinear et al., 2020), therapies that can support the patient to generate appropriate functional patterns of brain activity and motor behaviour are greatly needed, during the period when they are unable to generate actual movement unassisted.

## Brain-Computer Interface for Neurorehabilitation; Basic Premise and Scope of the Review

Non-invasive brain-computer (and brain-machine) interfaces provide an advanced technological solution, decoding brain signals directly from the scalp and translating them into movement of a virtual (on screen) or robotic effector. The effector can also be the user’s own limb, with electrical stimulation of muscles triggered by brain activity (Biasiucci et al., 2018; Bai et al., 2020). By closing the disrupted sensorimotor loop and providing tangible feedback, the patient learns to control the effector by motor imagery or movement intentions. Restoring relevant sensory feedback in relation to volitional movement attempts is believed to mobilise the fundamental mechanisms of motor learning (Mrachacz-Kersting et al., 2021). As such, they can engage in mental practise of movement and keep their motor neural circuitry active, warding off the detrimental effects of limb non-use (Buxbaum et al., 2020), its associated white matter degeneration (Egorova et al., 2020), and promote use-dependant neuroplastic processes (Xing and Bai, 2020). Despite largely encouraging evidence suggesting that functional gains are produced exceeding those of standard care (Hatem et al., 2016; McConnell et al., 2017; Monge-Pereira et al., 2017; Cervera et al., 2018; López-Larraz et al., 2018; Raffin and Hummel, 2018; Carvalho et al., 2019; Coscia et al., 2019; Kovyazina et al., 2019; Remsik et al., 2016; Xing and Bai, 2020) substantial challenges with existing BCI implementations have prevented widespread adoption of the technology clinically (Baniqued et al., 2021). Here we provide a viewpoint on the practical, technical and mechanistic factors impeding the scalability of BCI into rehabilitative care packages. Further, we provide a future outlook for optimal design of the technology to best exploit its unique potential, and best practices for research and reporting of findings.

Non-invasive BCI typically consists of three key components: A recorder, a decoder, and an effector. The recorder acquires brain signals from the scalp surface. The decoder analyses the recorded data, and the effector acts upon the information. In most cases the recorder is an electroencephalogram (EEG) detecting scalp electrical fluctuations associated with neuronal activity. In practise, any neural signal could be incorporated into a BCI, and implementations using magnetoencephalography (MEG) (Buch et al., 2008; Foldes et al., 2015), functional magnetic resonance imaging (fMRI) (Thibault et al., 2018) and functional near-infrared spectroscopy (fNIRS) (Soekadar et al., 2021) have also shown merit. The decoder is usually a programme run on a computer, extracting desired aspects from the signal and conducting analyses in real-time. The analysis process may be as simple as measuring amplitude or frequency of ongoing brain activity (Wierzgała et al., 2018), or more complex decompositions of inter-regional connectivity or dynamic changes to spatial patterns of activation (Rathee et al., 2017). The effector of a BCI can take multiple forms. For neurorehabilitation, it may be a device that assists the patient to complete movements, such as a robotic limb (Tariq et al., 2018; Soekadar et al., 2019; Khan et al., 2020), a device that gives virtual (e.g., on-screen) feedback to the participant to promote appropriate patterns of neural activity (Kerous et al., 2018; Si-Mohammed et al., 2018; de Castro-Cros et al., 2020), or a trigger to induce electrical stimulation of muscles in order to evoke movement (Biasiucci et al., 2018; Bai et al., 2020). Even in the absence of evoked movement, electrical stimulation can be used below motor threshold to provide continuous somatosensory feedback as the BCI signal (Corbet et al., 2018).

## Practical and Technical Challenges With Clinical Implementation of BCI

For the BCI participant, learning to control the effector requires multiple practise sessions, viewing continuous feedback and learning by reward (Chavarriaga et al., 2017; Mrachacz-Kersting et al., 2021). While passive/implicit learning is known to play a role in BCI control (Othmer, 2009), most human participants report developing and fine-tuning mental strategies throughout the course of training, usually involving imagination of movement (Majid et al., 2015; Ruddy et al., 2018; Khan et al., 2020), or in the case of brain injured patients, attempts to make movement with the paretic limb (Blokland et al., 2012; Balasubramanian et al., 2018; Bai et al., 2020). Even for neurologically healthy participants, gaining effective control of an EEG-BCI takes many distinct sessions (Pfurtscheller et al., 2003; Stieger et al., 2021). Without seeing tangible results within the first training sessions, it is likely that patients loose motivation to continue investing effort into trying to control the BCI. Another factor known to influence learning is the large variation in individual capability for motor imagery, contributing to the fact that 10–30% (Allison and Neuper, 2010; Vidaurre and Blankertz, 2010; Lotte and Jeunet, 2015) of users never achieve control over the BCI; a phenomenon historically referred to as BCI illiteracy but more recently coined BCI inefficiency (Thompson, 2019). For BCI to serve as a useful therapeutic tool for neurorehabilitation, solutions that allow users to achieve control within a shorter time frame, and that are effective across a wider range of motor imagery capabilities, are needed to secure the future of this technology.

As the neural signals used to drive effectors in an EEG-BCI are heavily influenced by ongoing mental state, the individual capacity to generate high quality mental imagery of movement dictates how easily detectable the relevant motor signals will be (Marchesotti et al., 2016; Chavarriaga et al., 2017). Visual motor imagery, imagining observing yourself perform a movement, produces less pronounced scalp-recorded signals than kinaesthetic motor imagery where the feeling of movement is effectively experienced (Neuper et al., 2005). Kinaesthetic motor imagery produces more easily detectible neural signals, at scalp locations overlaying motor cortical brain regions, and additionally modulates corticospinal excitability measured *via* motor evoked potentials (MEPs) in response to transcranial magnetic stimulation (TMS) (Stinear et al., 2006). However, about half of participants find it difficult to perform kinaesthetic motor imagery (Seiler et al., 2017), and those are the lowest performers on BCI (Vuckovic and Osuagwu, 2013). In some circumstances, it may be more beneficial to request that the patient make attempts to execute movements, rather than simply imagine movement (Blokland et al., 2012; Balasubramanian et al., 2018; Bai et al., 2020). For motor imagery-based BCI, multimodal or multi-phase BCI approaches (Leamy et al., 2011; Fazli et al., 2012) may lead to better accuracy, as different neuroimaging modalities may be more sensitive than EEG to detect very weak motor signals (albeit, technologies such as MRI, fNIRS, MEG, or TMS may be less practical for long-term practise of BCI). As an example of a potential multi-phase approach, using a BCI based not upon scalp signals but upon muscle responses to TMS over the motor cortex, control of on-screen feedback using motor imagery could be achieved within just two training sessions (Ruddy et al., 2018). Whereas EEG scalp signals associated with movement intentions have poor spatial resolution, TMS can be used to target the motor cortical representations for specific muscles, selectively providing feedback of excitability of corticospinal pathways by measuring the amplitude of motor evoked potentials (MEPs) recorded at the muscle with electromyography (EMG). When tested in a sample of stroke patients, most were capable of learning to increase the excitability of their corticospinal pathways with TMS neurofeedback, using only motor imagery (Liang et al., 2020). Using a multimodal approach it may be possible to train individuals to develop robust motor imagery strategies that optimally excite the targeted brain-muscle pathways using TMS neurofeedback within just two sessions, that then translates to better subsequent performance using functionally relevant signals on an EEG BCI that has better portability options. This hypothesis remains to be tested empirically, and it is notable to point out that the approach may only be applicable to patients who exhibit MEPs when stimulated. Approximately 13.4% (Stinear et al., 2017a) are deemed “MEP negative,” and those tend to be the most severely affected (Stinear et al., 2017b; Smith et al., 2019; Lundquist et al., 2021). Incorporating multimodality into BCI paradigms may also extend beyond the aforementioned suggestions concerning acquisition modalities. Multimodal feedback (i.e., visual plus auditory or somatosensory) can also enhance the BCI learning experience (Sollfrank et al., 2016) and improve the quality of detectable brain signals (Sollfrank et al., 2015).

In order for BCI for neurorehabilitation to become scalable, it needs to answer to the current requirements of healthcare providers. Namely, it should reduce rather than increase burden and need for expert supervision, and instead place high quality rehabilitation into the hands and home of the patient in a cost effective manner. Even if initial control of the EEG BCI can be achieved more quickly using multimodal or multiphase approaches, longer term use over weeks or months would still be required alongside the patient’s standard rehabilitative care in order to promote functional upper limb improvement. Current implementations of EEG-BCI are not well adapted for this purpose as they are cumbersome, require lengthy setup times with wet electrolyte-filled sensors, and a skilled operator to ensure sufficient signal quality, positioning of the headgear and execution of (often custom written and not user friendly) software. Recent technological advances in wireless, high impedance (dry electrode) EEG systems may enable better scalability. Using tablet-based software allowing real-time wireless streaming from a comfortable, wearable EEG cap with dry electrodes, signals of sufficient quality can be recorded even in home environments by elderly participants without assistance (Murphy et al.,2018a,b, 2019; McWilliams et al., 2021). Advancing this new technology to additionally provide real-time feedback to the participant is a necessary next step towards home-based BCI that would allow extended training to be conducted in the weeks and months following brain injury. Further, it encourages the patient to feel in control of their own recovery process, rather than dependant on professionals or their caregivers. To date, existing implementations of wireless BCI for neurorehabilitation are at an early stage of technology readiness, with none reaching even small scale clinical trials (Baniqued et al., 2021).

## Challenges for Elucidating Mechanisms Underlying BCI-Induced Functional Improvements

Advocating for clinical use of BCI is difficult when the specific mechanisms underlying functional improvements remain largely unknown. In order to make justifiable predictions regarding whether a patient is likely to benefit from BCI training, clinicians need to know what aspects of neural function are being targeted. The vast heterogeneity of available BCI methods further complicates attempts to elucidate mechanisms, as it is likely that different approaches target different aspects of neural circuitry to bring about functional improvements. A key issue to shed light upon across all types of BCI for neurorehabilitation is the potential contribution of unspecific (i.e., non-motor) mechanisms for recovery. Ros et al. (2020) name four non-specific factors that may contribute to overall BCI performance improvement. These include (i) factors unique to the neurofeedback environment (such as trainer-participant interaction in a neurotechnology context). (ii) Factors that are common across interventions (such as benefits from engaging in a form of cognitive training, as well as psychosocial and placebo mechanisms related simply to participating in an experiment). (iii) Repetition-related effects and (iv) natural effects occurring during the intervention period such as cognitive development (in children/adolescents) or age-related cognitive decline in elderly participants. This list is, however, not exhaustive. Additional to the aforementioned mechanisms, BCI performance may also be influenced by effects from sustained exertion of effort or engagement, feelings of empowerment or competence from achieving control of the BCI, or general improvements in mood or enjoyment resulting from engaging with a challenging gamified task. Even with seemingly adequate control groups, it may be challenging to match aspects such as effort, attention, enthusiasm, and enjoyment between those receiving real neurofeedback and those receiving pseudofeedback which may be less intrinsically motivating. While it is encouraging that many studies are now including explicit measures to monitor training-induced changes to motor neural circuitry (i.e., using neuroimaging), measures of the aforementioned unspecific effects are rarely included. Thus, their contribution cannot be evaluated with the currently available evidence.

While the presence of unspecific BCI effects makes it more difficult to draw conclusions on how motor improvement for neurorehabilitation is achieved, it leaves open the intriguing possibility that although BCI training is conducted in the motor domain (i.e., using motor imagery), beneficial effects may not be exclusive to the motor system. For instance, it is conceivable that increased effortful focus on the BCI task over a sustained training period may lead to a top-down, generalised improvement in brain health, materialising as motor gains (measured by most studies) but also gains in other (e.g., cognitive) domains, which are not routinely quantified in BCI studies. This may materialise in the form of improved executive function, memory, attention, or processing speed. Such general improvements may be brought about by, for example, increased blood flow to the brain, enhanced neurochemical environment promoting plasticity induction, or simply increased traffic in neural circuitry sustaining healthy activity-induced myelination processes. Generalised (cross-domain) transfer from trained to untrained tasks is greatest when the trained task requires a high degree of attentional focus and cognitive flexibility (Green and Bavelier, 2008; Bavelier et al., 2018). In this regard, it is debatable whether the motor imagery BCI is primarily a motor task, or a cognitive task, making direction of transfer difficult to ascertain. Future work may focus on whether motor improvements arise as a result of transfer from improved cognitive function, or whether a portion of the specific motor improvements transfer to improve cognitive function. To test this empirically, design of future BCI studies should routinely include cognitive performance measurements alongside motor function tests, with measures taken at multiple timepoints throughout the learning process.

A small proportion of randomised controlled trials (RCTs) investigating BCI for neurorehabilitation have made efforts to measure and/or discuss potential mechanisms that give rise to functional improvements. Of these, candidate neural changes that co-occur with improvement in motor function include enhanced desynchronisation of sensorimotor rhythms (i.e., neural oscillations in the alpha 8–12 Hz and beta 13–30 Hz frequency range) over scalp locations corresponding to motor cortex (Buch et al., 2008; Prasad et al., 2010; Li et al., 2014; Ono et al., 2015), changes in functional connectivity (Varkuti et al., 2013; Pichiorri et al., 2015; Biasiucci et al., 2018; Wu et al., 2020), lateralisation of neural activity (Ramos-Murguialday et al., 2013) or changes to white matter microstructure (Song et al., 2015; Hong et al., 2017). Others have speculated that BCI works by mobilising the brain’s intrinsic learning mechanisms, adapting behaviour using classical and/or operant conditioning giving rise to neural adaptations (Mrachacz-Kersting et al., 2021). To date, there has not been a comprehensive account that successfully resolves the aforementioned different perspectives into a holistic mechanistic model encompassing the electrophysiological, haemodynamic, and neurochemical components. Multimodal investigations measuring BCI-related neural changes simultaneously in each of these different modalities (e.g., EEG, fMRI, and MR-spectroscopy) are warranted in order to understand how the neural elements interact to bring about functional motor improvements. A point to note is that in none of the above studies were non-motor, unspecific mechanisms tested, so their contribution to improving motor function remains unknown. Transfer of benefits to the non-motor domain were also not quantified, leaving open the possibility that improvement in motor function may be a result of general brain health improvement.

Elucidating mechanisms of functional improvement from BCI is further complicated by the fact that brain injured patients have widely heterogenous lesions, and lesion size and location do not predict functional outcomes in a straightforward manner (Umarova et al., 2021). Even in patients with similar extents of impairment, lesion location influences performance of BCI decoding of movement intentions (López-Larraz et al., 2017), and the scalp detected signals are qualitatively different when comparing cortical vs subcortical lesions in particular (López-Larraz et al., 2019). This poses challenges for a “one size fits all” approach to BCI for neurorehabilitation and stresses the importance of adaptive algorithms that do not make rigid *a priori* assumptions regarding location or characteristics of scalp detected signals, but rather allow flexibility to detect idiosyncratic patterns of neural activity that could be used to drive the BCI in an individually tailored fashion.

## Outlook for Future Scalability and Justification of BCI Use Clinically

The field of BCI for neurorehabilitation has benefitted in recent years from collaborative efforts to standardise approaches using the best evidence-based technologies, and with recommendations for best practise in conducting research. For instance, the mental-task based BCI (MT-BCI) consortium is a multinational effort collecting the largest existing sample of BCI data across nine countries, with the objective to deepen understanding of learning mechanisms in MT-BCI, improve efficiency and reliability of MT-BCI and make them more useable for clinical and non-clinical applications (Jeunet et al., 2020). This “big data” approach to BCI breaks away from the typical small scale studies that are characteristic in this field, and may facilitate more advanced analyses techniques such as machine learning.

A key challenge is to make BCI tasks more user friendly, providing motivating feedback in a style that the user finds useful (Kübler et al., 2014; Pillette et al., 2017). Both hardware and software must be simple to use for patients and caregivers alike, which may result in greater enthusiasm towards the technology (Käthner et al., 2017). Tasks should avoid being fixed and repetitive, but rather should have an adaptive nature allowing the user to clearly see progression through stages as performance improves (Jeunet et al., 2016). BCI approaches that focus on assistance with activities of daily living (Soekadar et al., 2016) (particularly bimanual tasks in stroke patients) during physiotherapy may foster motivation and generalisation of skills towards everyday life (Soekadar et al., 2019). Additional to this, attempts to improve scientific rigour and reproducibility in neurofeedback research have established the CRED-nf framework for reporting of results, and recommendations for future design of studies (Ros et al., 2020). It is hoped that these collaborative efforts will improve understanding of BCI mechanisms by establishing a degree of standardisation of measurement, and ensuring that adequate experimental controls are in place.

## Conclusion

The technological and practical scalability and clinical justifiability of BCI still pose challenges preventing widespread use for neurorehabilitation. Recent advances in knowledge and technology provide opportunities to facilitate a change, provided that researchers and clinicians using BCI agree on standardisation of guidelines for protocols and shared efforts to uncover mechanisms. Addressing BCI inefficiency and speed of learning are priorities for the field, which may be improved by multimodal or multi-stage BCI approaches harnessing more sensitive neuroimaging technologies in the early learning stages, before transitioning to more practical, mobile implementations. Clarification of the neural mechanisms that give rise to improvement in motor function is an essential next step towards justifying clinical use of BCI. In particular, quantifying the unknown contribution of non-motor mechanisms to motor recovery remains elusive and calls for more stringent control conditions in experimental work. Measurement of additional neural (non-motor) systems and of performance on non-motor tasks is also essential to demonstrate specificity or transfer of the improvements across cognitive and motor domains. If the effects of motor imagery based BCI are found to generalise beyond the motor system, for instance to improve cognitive control or gait, the potential relevance of BCI is expanded presenting an intriguing opportunity for the field. Ultimately, if the benefits are further found to generalise beyond lab-based experimental settings to more ecologically valid aspects affecting quality of life such as competence and autonomy (Lövdén et al., 2010; Cremen and Carson, 2017), the intervention can truly be deemed as effective and worthwhile implementing clinically for neurorehabilitation.

## Author Contributions

CS wrote the first draft and edited the final draft. KR and CS conceptualised the idea for the manuscript. KR, SS, NK, and DB worked on subsequent drafts of the manuscript editing and providing feedback. All authors reviewed the final version and were involved in discussions throughout.

## Conflict of Interest

The authors declare that the research was conducted in the absence of any commercial or financial relationships that could be construed as a potential conflict of interest.
